# Character Strengths as Coping Strategies for Daily Challenges: A Qualitative Study Among Adult Refugees

**DOI:** 10.1007/s41042-024-00211-z

**Published:** 2025-02-08

**Authors:** Tom Hendriks, Jorien van Treeck, Ranim Chaya, Joop T. V. M. de Jong, Marianne van Woerkom

**Affiliations:** 1 GZA Healthcare, Utrecht, The Netherlands; 2 Department of Medical and Clinical Psychology, Tilburg University, Tilburg, The Netherlands; 3 Pharos, Utrecht, The Netherlands; 4 Tilburg University, Tilburg, The Netherlands; 5 Amsterdam UMC, Amsterdam, The Netherlands; 6 Department of Human Resource Studies & Department of Developmental Psychology, Tilburg University, Tilburg, The Netherlands

**Keywords:** Resilience, Refugees, Character strengths, Coping, Asylum center, The Netherlands, Hope, Perseverance, Gratitude, Self-regulation

## Abstract

Character strengths are positive traits and qualities that are an integral part of an individual’s personality. These strengths can function as protective factors against mental disorders by providing traumatized refugees with resources and tools to cope with stressors and daily challenges. The purpose of the current study was to explore the role of character strengths of refugees in the Netherlands in their problem-focused and/or emotional-focused coping strategies to deal with daily challenges. We interviewed 26 refugees who attained a temporary asylum residence permit and who lived in an asylum seeker center (AZC) or a municipality in the Netherlands. Qualitative data analyses yielded five key character strengths, namely self-regulation, perseverance, gratitude, love of learning, and hope. Patience and adaptability were two additional strengths that function as specific self-regulation techniques. Our findings also suggest a shift from emotion-focused coping strategies during the stay at an asylum seeker center towards problem-focused coping when living in a municipality. Depending on the migration phase in which refugees find themselves, they apply different character strengths in relation to emotion-focused or problem-focused coping strategies. Interventions that aim to increase the well-being of refugees and contribute to successful integration into society should consider relevant contextual factors, in addition to cultural factors.

**Supplementary Information:**

The online version contains supplementary material available at 10.1007/s41042-024-00211-z.

## Introduction

The contextual risk factors for mental health problems among refugees, as a result of traumatic experiences before, during, and after their flight to a host country, have been described in a myriad of studies. Risk factors include traumatic life events, such as exposure to life-threatening situations (Nakash et al., 2015), dire situations in transit camps (de Jong & van Dijk, 2020), and the uncertainty of not knowing whether one can remain in a host country (Ryan et al., 2008). In addition, mental health problems can be exacerbated by social isolation, constant concerns about family members left behind, perceived and real discrimination, and loss of social and economic status (Malm et al., 2020). These risk factors influence the possibilities to navigate daily challenges.

At an individual level, the impact of such risk factors on mental health may also depend on the coping styles and strengths of refugees. The *VIA Classification of Character Strengths and Virtues*, developed by Peterson and Seligman (2004), is a widely used taxonomy of character strengths. This framework identifies 24 individual strengths which reflect six cultural ubiquitous virtues (i.e., wisdom and knowledge, courage, humanity, justice, temperance, and transcendence). Examples of character strengths include creativity, zest, forgiveness, kindness, and gratitude. The VIA classification offers a framework that can be used to map-out personal strengths in a systematic and comprehensive way (McGrath & Walker, 2016).

Previous studies have associated character strengths with a wide variety of increased well-being-related outcomes among both healthy and clinical populations, including subjective well-being (Gillham et al., 2011), psychological well-being (Yan et al., 2020), resilience (Shoshani & Slone, 2016), and post-traumatic growth (Duan & Guo, 2015). However, literature on VIA character strengths among refugee populations is rare and– to our knowledge - includes only two studies. One correlational study on the relationship between psychological well-being and character strengths among a group of North Korean refugees who settled in South Korea reported small to moderate correlations between the character strengths courage, humanity, justice, temperance, transcendence, and wisdom on the one hand and psychological well-being on the other hand (Kim, 2019). Another study tested the effects of a six day multi-component positive psychology intervention among children in a Greek refugee camp, which included one sesssion that focused on understanding and using character strengths in general (Foka et al., 2020). This quasi-randomized pilot study reported increases in well-being, self-esteem, optimism, and a decrease in depressive symptoms in the intervention group compared with a wait-list control group. These findings underline the potential for more character strength-based research among refugee populations.

### Coping and Character Strengths

Coping is generally defined as the response to a stressful situation, involving the continuous changing of cognition and behavior, and managing specific demands of that situation (Lazarus & Folkman, 1984). In both the general population and among refugees specifically, employing adaptive coping strategies is linked to improved health outcomes (Seguin & Roberts, 2017). Conversely, the utilization of maladaptive coping strategies is predictive of adverse health consequences, including pain, depression, anxiety, and PTSD (Thompson et al., 2010; Woltin et al., 2018). While scholars agree upon the importance of coping, there is little consensus regarding its conceptualization, which has led to a wide array of classification of coping strategies. In the literature, there are over 400 categories and classifications that describe adaptive and maladaptive coping strategies (Skinner et al., 2003). An often-used categorization is the distinction between problem-focused and emotion-focused coping. Problem-focused coping involves managing the problems or stressors themselves, for example through problem solving or planning (Lazarus & Folkman, 1984). Emotion-focused coping involves managing the emotions related to the stressors (Baker & Berenbaum, 2007), such as seeking emotional support, emotional venting, and positive reinterpretation of events (Folkman & Moskowitz, 2004). While both coping styles are recognized as effective strategies, problem-focused coping is considered a more effective strategy (Carver & Connor-Smith, 2010), since emotion-focused coping strategies may include maladaptive ways of coping, for example avoidance and suppression (Schäfer et al., 2017). Both coping styles are also recognized in the literature pertaining to the field of refugee studies. For example, a study among 850 Syrian refugees reported that utilizing problem-focused coping strategies predicted greater post-traumatic growth (Ersahin, 2020). Likewise, a study among 335 refugees in the Netherlands found that emotion-focused coping and social support seeking (which is strongly related to problem-focused coping) had a direct positive effect on their quality of life (Huijts et al., 2012). In addition, a study among 409 Syrian refugees in Turkey underlined the importance of emotion-focused coping strategies for integration in their new home country, and concluded that both problem- and emotion- focused coping strategies are significantly hindered by traumatic experiences (Kurt et al., 2021). These studies confirm the positive effects of both problem-focused and emotion-focused coping strategies among refugees.

Whereas no previous studies have investigated the role of VIA character strengths in dealing with stress and trauma among refugee populations, numerous review studies have identified religiousness/spirituality (one of the 24 character strengths) as a major coping mechanism for refugees (Fadhlia et al., 2024). Religiousness provides a sense of coherence (Jeserich et al., 2023), offers structure, and cultivates a sense of control and meaning (Fernando & Ferrari, 2011). It can also contribute to accepting negative situations (Luster et al., 2009), and religious coping is associated with post-traumatic growth among refugees (Ersahin, 2020). Studies have consistently shown that the belief in a higher power, and the practice of rituals including meditation and prayer, has beneficial effects on the mental and physical well-being of refugees. A study among 4504 refugees living in Europe that tested the effectiveness of a spiritual education program revealed a significant decrease in trauma-related outcomes, and a significant increase in mental health (Pandya, 2018). This study also found positive effects of the intervention on optimism.

According to Peterson and Seligman (2004), hope and optimism embody a cognitive, emotional, and motivational approach to what lies ahead. Contemplating the future, expecting the realization of desired events and outcomes, and taking appropriate action is believed to be linked to the likelihood of a positive outcomes. Maintaining confidence that efforts will lead to positive results contribute to maintaining a positive outlook in the present moment and energizing actions aimed at achieving goals. Hope and optimism are intertwined strengths, with hope emphasizing emotional aspects, while optimism is centering on expectational aspects. Both are often mentioned as protective factors for refugee populations. Further more, a positive relation between religiousness and optimism/hope has been documented in myriad studies, for example among displaced refugee parents and their children from Syria (El-Khani et al., 2017). In contrast, hopelessness is often reported as risk factor for mental wellbeing (Renner et al., 2020).

In addition to religion/spirituality and hope/optimism as protective factors for refugees, we occasionally encounter gratitude, forgiveness, perseverance, and self-regulation as coping strategies in the literature, although to a much lesser extent. For example, Liu et al. (2020) reported that being grateful and recognizing the opportunity for a fresh start was a positive coping strategy for refugees from the Middle East and Africa, who had to cope with the stress of resettlement in Canada. Forgiveness may be another relevant strength in the context of well-being among refugee populations, as was suggested in studies among refugees and asylum seekers from countries including Ukraine (Skalski-Bednarz et al., 2024), Iraq (Kira et al., 2009), and Syria (Paloutzian & Sagir, 2019). Perseverance is a character strength that we occasionally encounter in the literature and mostly in the context of resilience. It is considered an essential personal strength for enduring traumatic pre-migration events including structural oppression and violence (Cigrand et al., 2022) and peri-migration stress factors including living in overcrowded refugee camps (Shepherd et al., 2020), and dealing with the lengthy asylum procedure in a host country (Walaardt, 2011). Finally, a character strength that we occasionally come across as a protective factor is self-regulation. For example, a study among Palestinian adolescent refugees suggested that high levels of self-regulation predicted resilience (Aitcheson et al., 2017). In addition, previous studies have identified self-regulation as a skill for unaccompanied refugee minors to deal with traumatic events (Huemer et al., 2011), a skill required for academic self-efficacy among Syrian refugee students (Sari et al., 2020), and for refugee career adaptation (Wehrle et al., 2019).

### Present Study

The present qualitative study explores character strengths as coping mechanisms of refugees in the Netherlands in navigating daily challenges, drawing on interviews with individuals currently residing in asylum centers and those previously in such centers who have since transitioned to living in Dutch municipalities. To provide context, we will first outline the phases refugees undergo when applying for asylum and then touch upon the challenges refugees are facing.

Upon arrival in the Netherlands, refugees report to the Aliens Police (AVIM) at designated application centers for identity verification and application submission. They are then offered temporary shelter by the Central Agency for the Reception of Asylum Seekers (COA). After medical screening, the Immigration and Naturalisation Service (IND) conducts initial interviews to gather background information. Next, asylum seekers enter a preparation period before undergoing detailed interviews in the general asylum procedure, where the IND assesses their eligibility for protection. Based on the outcome, they may receive a temporary residence permit (thereby becoming so-called status holders), a rejection, or an extended decision process. If granted a residence permit, they are assigned to a municipality responsible for their housing and integration. Rejected applicants typically have four weeks to leave the country voluntarily or may appeal the decision in court (COA, 2024).

In sum, refugees go through three phases: (i) the asylum application phase; (ii) the housing application phase; and (iii) the integration phase. During the first and second phase, when refugees are living at an asylum center, they face different challenges. These include prolonged uncertainty about legal status (Hvidtfeldt et al., 2020), family concerns and loneliness (Nowak et al., 2023), substandard housing conditions and limited privacy (Nassim et al., 2021), financial and material dependency (Malm et al., 2020), and difficulties related to lifestyle and dietary adjustments (Dharod et al., 2013). Once they entered the integration phase, where they live outside an asylum center, refugees will face other challenges including adjusting to the new culture, the climate, learning the Dutch language, and perceived discrimination (Fadhlia et al., 2022; te Lindert et al., 2008; Warfa et al., 2012). A significant obstacle is labor market integration, as refugees often face difficulties in securing employment that matches their qualifications and experience (de Vrome & van Tubergen, 2010). Research also indicates that refugees often experience heightened symptoms of post-traumatic stress disorder (PTSD) after transitioning from asylum centers to independent living situations (Knipscheer et al., 2015).

Previous studies have thus identified religiousness/spirituality and hope/optimism as the main coping mechanisms for refugees to deal with trauma and stress. In addition, gratitude, forgiveness, perseverance, and self-regulation are occasionally mentioned in studies that focus on protective factors for refugee well-being. However, the VIA classification includes 24 strengths, and this indicates that there might be a broader spectrum of character strengths that are helpful in developing adaptive coping strategies among refugees. Therefore, the central research question of the present study was: What character strengths contribute to adaptive coping with the daily challenges in the lives of status holders living at AZCs and during their integration in Dutch municipalities?

## Method

This article is part of a larger study on the cultural adaptation of a mental health prevention program called BAMBOO. This program aims to increase the resilience and mental well-being of refugees living at an AZC (Hendriks et al., 2024). For this study we used Template Analysis, a method that is used in qualitative research, characterized by the development of a coding template which represents themes identified. Template Analysis combines structured coding with the adaptability needed to capture the richness of qualitative data (Brooks et al., 2015). In reporting, we followed the Consolidated Criteria for Reporting Qualitative Research (COREQ) checklist (Tong et al., 2007). The COREQ is a widely recognized framework designed to enhance the quality and transparency of reporting in qualitative research. It consists of 32 criteria organized into three domains (i.e., research team and reflexivity, study design, and data analysis and reporting.Data collection was carried out in the period May to November 2021.

### Participants

In total, 26 candidates were approached face-to-face, by e-mail or telephone. There were no candidates who refused or dropped out, so in total 26 semi-structured interviews were conducted. Interviewees were recruited through purposive and snowball sampling among AZC residents who participated in the BAMBOO program, and through the personal network of the interviewers. The interviews were conducted by a team of six interviewers; 15 interviews were conducted face-to-face (58%), and 11 interviews were conducted online (42%). Six interviews were conducted by a Lebanese scholar (woman), three by an Iranian scholar (man), and 17 interviews were conducted by four Dutch scholars (three women and a man), with the (online) assistance of a professional interpreter or the Lebanese and Iranian scholar. Nine interviews were conducted in Arabic, six interviews in English, five in Farsi, four in Dutch, and two in Tigrinya. Interviewers followed an interview protocol (see Supplemental material 2) that was created by the main researcher and interviewers were instructed how to conduct the interviews. All interviewers were working in the field of refugee studies at the time of the interviews. Interviewers were acquainted with 18 interviewees, due to their participation in the BAMBOO program prior to the interviews, or because interviewees were recruited through the personal network of the interviewers. The interviews lasted between 45 and 60 min. Interviews with AZC residents were interviewed at the asylum center where they resided, interviews with participants residing in a municipality were interviewed online. There were no repeat interviews. Interviewees characteristics are shown in Table 1.

**Table 1:** Interviewees characteristics (*n* = 26)

Residing at AZC						Residing in a municipality				
#	Gender	Age	Country	Religion		#	Gender	Age	Country	Religion
1	Woman	46	Turkey	Islam		15	Woman	19	Turkey	Islam
2	Woman	38	Uganda	Christian		16	Woman	27	Syria	Druze
3	Woman	32	Yemen	Islam		17	Woman	28	Syria	Not religious
4	Woman	39	Eritrea	Christian		18	Woman	38	Syria	Islam
`5	Woman	34	Iran	Christian		19	Woman	33	China	Not religious
6	Woman	31	Eritrea	Christian		20	Woman	36	Syria	Islam
7	Man	39	Yemen	Not religious		21	Woman	29	Afghanistan	Christian
8	Man	41	Yemen	Islam		22	Man	26	Syria	Islam
9	Man	35	Iran	Islam		23	Man	27	Iran	Christian
10	Man	34	Sudan	Islam		24	Man	36	Iran	Islam
11	Man	62	Syria	Islam		25	Man	30	Eritrea	Christian
12	Man	57	Egypt	Islam		26	Man	39	Iran	Yarsan
13	Man	30	Afghanistan	Islam						
14	Non-binary	29	Jordan	Not religious						

### Procedure

Interviewees were informed of the general goal of the study (e.g., ‘identify character strengths that help you in dealing with stress and challenges in your daily lives’). The main interview topics were stress/daily challenges and well-being, character strengths at an AZC, and character strengths in relation to life in a Dutch municipality. Strengths were identified according to the VIA Classification of Character Strengths by using a card set that depicted a symbol of each strength and the name of the strength in Dutch, English, French, Arabic, Farsi, and Tigrinya (Hendriks et al., 2024). As critics of the VIA classification of character strengths have noted, the VIA framework primarily aligns with Western, individualistic constructs of virtues, limiting its global relevance and applicability (Chistopher & Hickenbottom, 2008). Therefore, we included two additional strengths (i.e., acceptance and harmony), which may be crucial as coping strategies among non-Western populations (Hendriks et al., 2018) and two strengths (i.e., patience and adaptability) that may be highly relevant for refugees (Nickerson et al., 2022; Walaardt, 2011).

AZC residents were asked to choose five to seven character strengths cards they found important in dealing with challenges at an AZC, as well as character strengths they thought they would need during their life outside an AZC. Similarly, interviewees already living in a municipality were asked what character strengths they currently found useful, and to reflect on the character strengths that helped them cope during their time at an AZC. The main researcher was provided with field notes from the interviewers via e-mail.

### Data Analysis

All interviews were audio recorded and then transcribed verbatim in English or Dutch and coded in English. Data that could lead to the identification of the interviewees were omitted to ensure confidentiality. Due to limited available time and budget, back-to-back translation was not conducted, nor were transcripts returned to participants for comments or correction. During the process of data analysis, the first and second author discussed data saturation and reached consensus. After analysis of approximately 18 interviews, we found that interviews did not yield new themes, that there was a redundancy in the responses and that the data no longer provided richness and depth. Coding was conducted by the first and second author of this paper. Coding went through a three-stage procedure. In the first stage, we used a priori codes, namely the 24 VIA character strengths plus four additional strengths, as described in the previous section. These were split in two categories related to the context of an AZC or a municipality (e.g., ‘8. AZC-Honesty’– and ‘8. MUN-Honesty’). In addition, the codes ‘problem-focused coping’ (PFC) and ‘emotion-focused coping’ (EFC) were created and added to each of the character strengths quotes. Furthermore, the first and second author performed an open coding analysis for all interviews. In the second stage, axial coding was performed, where codes were combined into broader themes, and given a particular colour. In the third stage, selective coding was performed by the third author on two categorie i.e., interrelated character strengths and maladaptive coping strategies). Using a preexisting coding scheme can bias the open coding analysis in a qualitative study by introducing preconceived notions that shape how data is interpreted, potentially limiting the inductive nature of the process (Braun & Clark, 2006). In order to minimize the risk of bias during the open coding process, the first and second authors independently coded the data using the open coding method, alongside referencing the a priori framework. During and after completing independent analyses, coding was compared, and discrepancies were resolved through discussion. This process ensured that open coding remained grounded in the data and not overly constrained by the preexisting framework. We also triangulated findings across multiple interviews to validate themes and ensure they emerged consistently across different contexts, rather than being driven by a priori expectations. The process was conducted in iterative cycles, where the first and second authors continuously revisited the data and revised codes to ensure alignment with participants’ voices rather than preconceived notions.

Signature strengths are strengths that are central to one’s identity and are naturally expressed in daily life, contributing to personal fulfillment and well-being (Schutte & Malouff, 2019). Research has suggested that most people have around three to seven signature strengths (Peterson & Seligman, 2004). However, in this study we will focus on key strengths, which we will desribe as an individual’s primary abilities, skills, or competencies that are critical for coping and achieving goals in specific contexts. Unlike signature strengths, these can be skills that are acquired, learned, or emphasized based on situational needs.

Analysis was conducted with the use of software program ATLASti version 9.1. IBM SPSS statistics v. 28.0 was used for the processing of the demographic data and the selected character strengths cards.

## Results

### Interviewees Characteristics

Twelve respondents identified as men, thirteen as women, and one as non-binary. The mean age was 35.2 years (SD = 9.2) with a range of 18-61 years. Participants originated from Afghanistan (2, 7.7%), China (1, 3.8%), Egypt (1, 3.8%), Eritrea (3, 11.5%), Iran (5, 19.2%), Jordan (1, 3.8%), Sudan (1, 3.8%), Syria (6, 23.1%), Turkey (2, 7.7%), Uganda (1, 3.8%) and Yemen (3, 11.5%). All 26 refugees were status holders, 13 lived at an AZC (50%) and 13 obtained a residence in a Dutch municipality (50%).

### Key Character Strengths

**Fig. 1:**
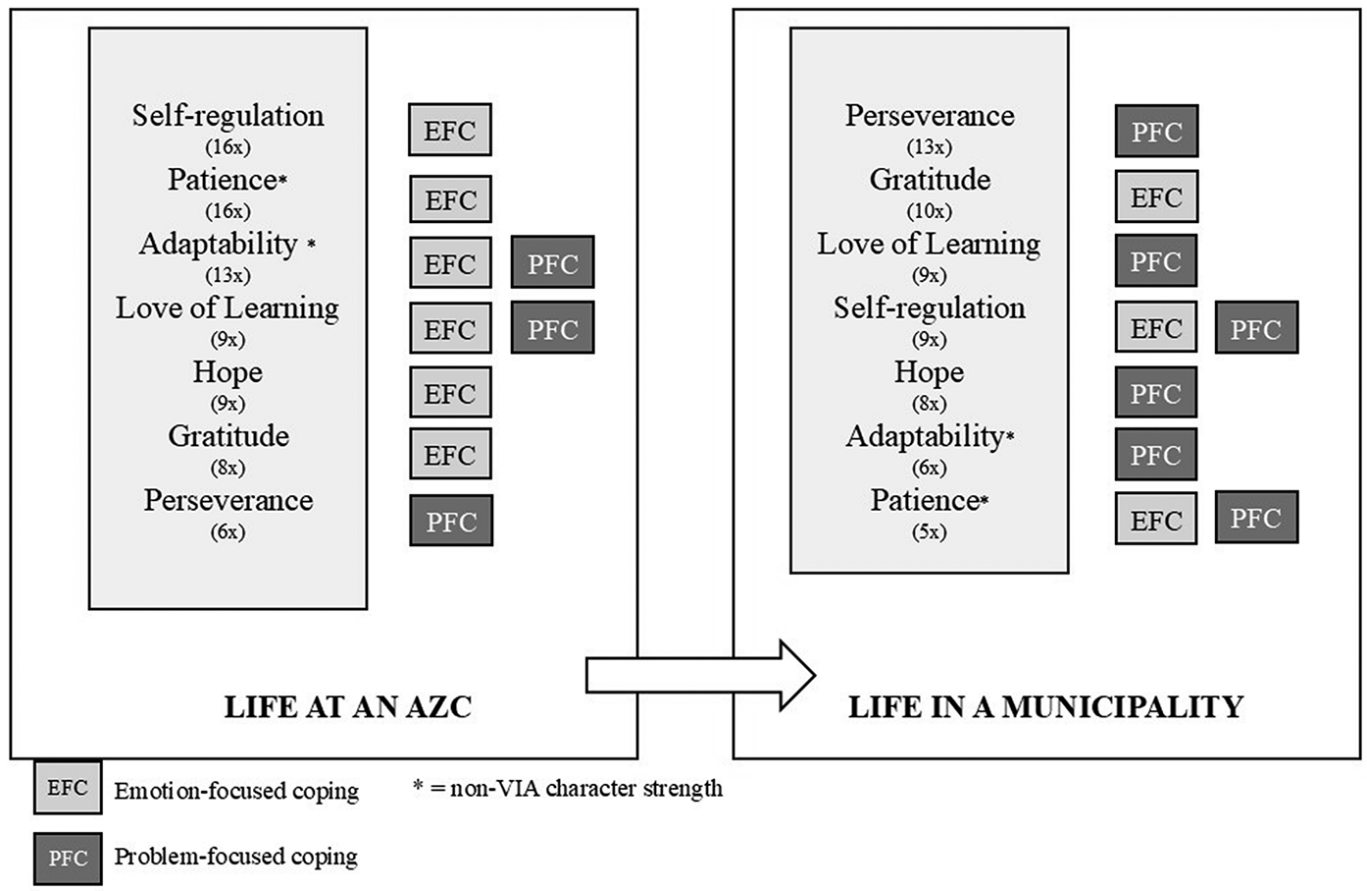
Summary and visualisation of the main findings

In total, we identified seven key character strengths - character strengths that are essential for adaptive coping, according to our respondents. These included five VIA character strengths, namely self-regulation, perseverance, gratitude, love of learning and hope, and two non-VIA character strengths, namely patience and adaptability. Our findings suggest a shift from emotion-focused coping strategies during the stay at an asylum seeker center towards problem-focused coping when living in a municipality. Figure 1 provides a visualisation of these seven key strengths, their mentioned frequency, and indication of what coping strategies they were primarily associated with life at an AZC and in a Dutch municipality.

A complete overview of the frequency of all selected character strengths can be found in Supplemental material 1. We will discuss each VIA character strengths and the two non-VIA character key strengths, and their relation to the coping strategies.

### Self-Regulation

Self-regulation was viewed as the most important character strength in dealing with challenges related to living in an AZC. According to our interviewees, self-regulation was needed to regulate negative thoughts—for example, refraining from constantly thinking back of traumatic events that happened in the recent past, or not allowing anger or negative thoughts to carry one away. Secondly, self-regulation was described as a strength that was needed for the discipline to spend the day productively—such as by walking, exercising, studying, and reading—to maintain physical and mental well-being. Thirdly, interviewees indicated that self-regulation was important to remain calm and respectful when communicating with others, even in stressful situations. Thus, in the context of an AZC, self-regulation can be primarily seen as an emotion-focused coping strategy.

“You need to control yourself. (…) Just trying to keep calm. If I let myself feel stressed or bad, I’ll make it worse for myself. So, I try to self-regulate and control that”, a 34-year-old man from Sudan.

In addition to its perceived function at an AZC, self-regulation was also frequently mentioned as a character strength that was required to cope with challenges in the daily life in a Dutch municipality. However, in that context, the emotion-focused and problem-focused coping functions were more equally balanced; both were deemed equally important. In addition to the role of self-regulation to cope with negative thoughts and emotions, interviewees also described it as a strategy to avoid problems in the integration process and as a way to attain the goals they had set in their new lives, as illustrated by the following quote:

“If there is no self-regulation in your work, you will not be successful in anything. I think everything I did, I did with self-regulation, so I was able to succeed”, a 29-year-old woman from Afghanistan.

### Perseverance

In order to deal with practical problems during their stay at an AZC, perseverance was a frequently selected character strength. Furthermore, it was the most selected character strength for dealing with challenges in life in a Dutch municipality. According to the interviewees, perseverance was necessary to deal with practical problems in the integration process into Dutch society. It was seen as a future-oriented personal quality, which was necessary to achieve goals—goals for the interviewees themselves, as well as for their families. One of our respondents voiced this in the following way:

“Perseverance is essential, to always push you towards your goals, not to have laziness when you have a goal and to persevere a lot when you have this goal”, a 41-year-old man from Yemen.

In particular, it was indicated that perseverance was necessary in order to learn the Dutch language. Perseverance was also deemed important to find work, perform a job, and deal with discrimination. Overall, perseverance was perceived as necessary to succeed in a Dutch municipality and can primarily be seen as a problem-focused coping strategy for life inside and outside an AZC.

“Perseverance is very important for a person who wants to be successful in society (…). I can say if you want to make progress in life you must have perseverance” a 29-year-old woman from Afghanistan.

### Gratitude

Gratitude can be seen as an emotion-focused coping strategy for living life at an AZC, as well as for living in a Dutch municipality. Most notably, being grateful was a way to be able to remain in a positive mood. A grateful attitude positively influenced the way one thinks and feels. Gratitude was expressed for, for example, having food, a bed, a safe place to sleep, or getting the opportunity to build a new life.

“In my opinion, being thankful for everything helps you to avoid nagging, being demanding and hurting yourself mentally”, a 39-year-old woman from Eritrea.

Gratitude was also related to the character strengths hope and perseverance; gratitude gives people the strength to perceive a positive future as well as the motivation to strive for positive goals. Gratitude was also strongly related to religion and spirituality. Many interviewees emphasized how important expressing gratitude towards God was for them. They asked God for protection, strength, and help, and when that came, they thanked God for it. Expressing gratitude on a daily basis was something most Islamic interviewees already did in their daily prayer; one respondent specifically referred to the often-used expression *subhanallah*– all glory goes to God. Some interviewees also expressed gratitude to the Dutch government in the interviews.

### Love of Learning

Love of Learning appeared to have three functions. Firstly, love of learning was considered to be a strength needed to learn the Dutch language, which was a struggle for all of the interviewees, regardless of their living situation. Secondly, love of learning was required to learn about Dutch culture and was viewed as an essential strength in the process of integration into Dutch society. Both functions can be related to problem-focused coping. Thirdly, in the context of life at an AZC, it was also described as a way to reduce emotional distress, a strategy to cope with worrying about the future and to avoid thinking about the traumatic experiences from the past. Learning new skills was also mentioned as a way to cope with loneliness and boredom, and a way to increase self-worth and experiencing positive emotions. These three functions are illustrated by the following two quotes of two of our respondents:

“In my opinion, it is important for two reasons. The first reason is that when you learn something new, you communicate better with people whose language you do not understand. (…) The second one is, when you want to learn something, if your mind is focused on learning, then your mind will not be distracted by your misfortune”, a 29-year-old woman from Afghanistan.

“Love of learning is very important; wherever we are we should always learn to improve ourselves. This is a good exercise for the brain not to be in sickness”, a 46-year-old woman from Turkey.

In the context of life in a municipality, the emotion-focused coping role of love of learning was not often mentioned.

### Hope

In relation to life at an AZC, hope was primarily described as an emotion-focused coping strategy. Maintaining hope was required in order to stay resilient. For example, it helped interviewees to get through the asylum procedure during their stay at an AZC, which they described as a stressful period. Interviewees often had to cope with the feeling of loneliness and had to remain hopeful about reunification with their loved ones in the future.

“You have to be optimistic, think tomorrow is a better day, everything will be fixed, everything won’t last forever. It is the reason you wake up every day. If you don’t have it, you can’t do it”, a 38-year-old woman from Uganda.

Furthermore, it was seen as a personal quality necessary to move forward in life. Hope motivated and gave people the energy to set future goals and to work towards them. This problem-focused coping approach was primarily voiced by interviewees when talking about strengths they needed in life in a Dutch municipality, as illustrated by the following quote:

We came here for the future of our children (…) I became aware about my rights and my children’s rights. All these made me hopeful and made me think positively. I thought in the future I will have a good future, and my life will be better than the life of many women who live in Afghanistan. Here, I can achieve my goals. These things made me feel positive about the future”, a 29-year-old woman from Afghanistan.

### Non- VIA Character Strengths: Patience and Adaptability

The two non-VIA character strengths that we included in this study (i.e., patience and adaptability) were both selected over ten times: According to our interviewees, patience was an essential strength needed to cope with the emotional challenges of daily life at an AZC. Some interviewees linked patience to dealing with feelings that are a result of dealing with the daily hassles and difficult living conditions at an AZC, such as anger, boredom, and frustration.

” If you are not patient, you will explode”, a 39-year-old man from Yemen.

Furthermore, patience was viewed as essential because the procedure of attaining a residence permit can be lengthy and unclear. During this process, patience was often put to the test, such as when refugees saw that other people at an AZC who came later to the Netherlands received their residence permit or obtained an external residence earlier than they did. Furthermore, waiting for reunification with one’s spouse and/or children required patience. Hence, in the context of life at an AZC, patience can primarily be viewed as an emotion-focused coping strategy. Regarding the role of patience in life in a Dutch municipality, patience was described as a problem-focused and an emotional-focused coping strategy because refugees must gradually get accustomed to Dutch social rules and legal regulations, as well as Dutch culture. In addition, goals—such as finding appropriate work or a steady, full-time work contract—cannot always be achieved as quickly as people would like.

Adaptability was a character strength that was selected by half of the interviewees in regard to dealing with daily hassles at an AZC, such as having to live together with other people in one room and having to share cooking and bathing facilities. It can be seen as a skill that was used to deal emotionally with several problems that arise from the challenging living conditions at an AZC. In this sense, adaptability can primarily be seen an emotion-focused coping strategy, and secondarily as a problem-focused coping strategy. This second aspect was vividly described by one our respondents in the following way:

“You will lack privacy for a long time (…). Sometimes you will be placed in shitty rooms and also a horrible camp, a far location, with old facilities. You do not get to choose. No matter how much you complain, it is pointless. You have to adapt”, a 28-year-old woman from Syria.

Regarding life outside an AZC, adaptability was largely described as a problem-focused coping strategy. One must adapt to the social and legal rules, customs, and traditions of Dutch society, which can be challenging. Examples given were being strictly on time for appointments, familiarizing oneself with Dutch laws and the bureaucratic system, and having to adapt to the Dutch climate.

## Discussion

In this study, 26 refugees were interviewed about the function of character strengths as strategies used to cope with daily challenges during their stay at a Dutch asylum center (AZC) and/or in a Dutch municipality. Qualitative analysis of the interview transcripts revealed five VIA character strengths that seemed to play a key role in adaptive coping, namely self-regulation, perseverance, gratitude, love of learning, and hope. In addition, two non-VIA character strengths were frequently selected as key strengths, namely patience and adaptability. The findings indicate that during the stay in an AZC character strengths were mainly used to deal with the emotional consequences of daily hassles and negative feelings that arise from traumatic experiences from the past (emotion-focused coping). In the context of life in a municipality, character strengths were also used to cope directly with current problems and not only their emotional consequences (problem-focused coping). In addition, character strengths seemed to be a motivational factor in working towards future goals. We found that self-regulation was viewed as the most important character strength, which played a key role in problem-focused strategies and, although to a lesser extent, in emotion-focused coping strategies. Our findings on the important role of self-regulation as a coping strategy are in line with findings in the general literature (Hagger et al., 2017), as well as in studies among refugee populations. For example, a qualitative study among German refugees reported that emotion regulation fostered adaptive coping by empowering refugees to engage to goalsetting in the context of career construction (Wehrle et al., 2019). Self-regulation may also play an important role in educational achievement among refugee students and youths (Sari et al., 2020). According to a study among 74 Afghan refugees (Koch et al., 2020), difficulties in emotion regulation accounted for significant variances in levels of PTSD, depression, and anxiety. Hence, it was suggested that emotional self- regulation is a transdiagnostic key factor in the prevalence of mental disorders. The second most frequently selected VIA character strength was perseverance. In the literature, perseverance has been described as an essential personal attribute that is used throughout all stages of the migration process (Cigrand et al., 2022). In our study, interviewees foremostly described perseverance in a future-orientated manner, considering it as a strength that was required to attain goals during the integration process into Dutch society.

Gratitude was the third most frequently selected VIA character strength. Our interviewees expressed their gratitude for the things that they currently had in their lives, for example a safe environment, food, shelter, and physical health. To remain in a positive mood, they were counting their blessings instead of focusing on their hardship and the things they lost in their lives. Generally, gratitude is seen as emotion-focused coping strategy (Wood et al., 2007). During our interviews, it became apparent that our interviewees felt indebted to the Dutch government and Dutch people in general. Expressing gratitude was a way for them to compensate for what their host country has done for them. Our findings also suggest that refugees express their gratitude towards God as an emotion-focused coping mechanism, which corresponds with findings from numerous studies that have identified gratitude towards God as major coping mechanism. For example, a qualitative study among 29 displaced refugee parents from Syria identified gratitude as a way that enabled them to focus on the good things they had despite their situation by being thankful towards God because their children were still alive and relatively healthy (El-Khani et al., 2017).

We identified love of learning as the fourth most frequently mentioned VIA character strength. It was mainly used as a problem-focused coping strategy. Our interviewees indicated that learning was an important requirement if they wanted to stay in the Netherlands and build a new life. This corresponds with the literature that recognizes learning as a crucial component in the transition to a new life in a host country, which not only requires the ability to learn new skills or another language, but also to acquire intercultural competencies (Hammer et al., 2003). For our interviewees, learning the Dutch language was a source of stress, and love of learning was a character strength that helped them dealing with this stressor. Our interviewees also identified love of learning to be important for acquiring knowledge about Dutch culture, Dutch legislation, and the cultural and social norms of Dutch people. Learning was also described as an emotion-focused coping strategy; it is a way to shift away the attention from past negative experience to more positive future expectations. Furthermore, learning was described as a pleasant experience and a way of self-expression. This view on learning activities is in line with findings from a study among Syrian refugees in Lebanon and Germany (Abou-Khalil et al., 2019), who described learning as a way to appreciate their identity, to fulfill the need for self-expression, and the need for entertainment and having fun. The fifth most frequently mentioned VIA character strength was hope. Our findings suggest that hope can be viewed as an emotion-focused coping strategy used to remain positive in the face of the uncertainty associated with the long asylum procedure during their stay at an AZC and as a problem-focused coping strategy by providing the motivation to achieve future goals. This is in line with a meta-ethnography study among young refugees—including 26 studies—which described hope as one of six sources of resilience (Sleijpen et al., 2016). A study among Sudanese refugees residing in Australia identified hope as a coping mechanism that could reframe negative experiences from past and present challenging situations (Khawaja et al., 2008). Similarly, a study among Syrian refugees listed hope as a personal coping strategy that was facilitated by the social network of refugees and the activities that were conducted with others (Renner et al., 2020). Faith in a higher power seemed to be an important source of hope for many of our interviewees. Our finding that religious belief can function as a source of hope is in line with other studies, including a study among Syrian refugees that concluded that religion was a source of happiness and hope among their interviewees (Karaman et al., 2022). A noteworthy finding in our study was that the card depicting the character strength religion/spirituality was not often selected by our interviewees (in total only twice, see Supplemental material 2). This was surprising, considering that many studies that have described the central role of religion and spirituality in the lives of refugees (Jung, 2015; Pandya, 2018). However, the supportive role of religion was often mentioned, and many interviewees expressed gratitude towards God during the interview. Possibly, our interviewees considered religiousness/spirituality not as a human character strength, but rather a strength that transcends personality. As a previous study on character strengths among a non-Western population suggested, religiousness may be considered the bedrock for character strengths among people from non-Western origins (Hendriks et al., 2018) rather than a distinct character strength.

Among the most selected character strengths were two non-VIA strengths. Firstly patience, which according to our interviewees could be seen as a self-regulation technique. Our interviewees indicated that patience was essentially needed to deal with the frustrations that emerge from the long asylum procedure, which can take several months and even years. This is consistent with other studies which have shown that refugees tend to regard patience as a coping strategy to deal with factors that have a negative impact on their health and well-being (Laban et al., 2008). The view of patience as a self-regulatory technique is also consistent with the description of patience by Peterson and Seligman (2004), two of the creators of the VIA Classification of Character Strengths, who described patience as a strength that melds character strengths self-regulation, persistence, and open mindedness.

Adaptability was another non-VIA character strength that was frequently mentioned by our interviewees, mainly in relation to coping with the daily hassles in life at an AZC and for instance the lack of privacy which is a major stressor in the lives of many refugees (Somasundaram, 2010). Adaptability refers to the capacity of an individual to regulate one’s cognition, emotion, and behaviour in situations of change, novelty, and uncertainty (Martin et al., 2012). Hence, adaptability can also be seen as a specific self-regulation technique. Our interviewees mentioned the importance of continuously adapting to the lack of privacy and having to share their room, kitchen, and bathroom facilities with other people.

### Limitations

Findings from this study should be understood within the context of the study’s limitations. Firstly, our study focused on strengths that were used by refugees who were living at an AZC or a municipality. It did not consider a range of influencing factors, including country and region of origin, gender, religious affiliation, time of arrival, and duration of their stay. Furthermore, we interviewed interviewees from eleven different countries, with an over representation of refugees from Syria (*n* = 6) and Iran (*n* = 5). This may limit the ability to generalize our findings to the larger population of refugees living at AZCs, that consist of people originating from over 100 countries. Secondly, this study was conducted during the COVID-19 pandemic in the Netherlands (May 2021 - November 2021), which limited the possibility to approach candidates at an AZC or in a Dutch municipality. For this reason, we used convenience sampling, instead of random sampling. Thirdly, the interviewees were self-selected by the interviewers and the ones who volunteered may hold stronger opinions compared to the general refugee population. However, the selective sampling may have also contributed to less bias. Eight out of the 26 interviewees had participated in the BAMBOO program and were already familiar with the concept of character strengths due to their participation in the program. This may have contributed to a deeper understanding of the topic among these interviewees than the ones who did not participate in the BAMBOO program, and hence have influenced their answers. In addition, ten of the interviewees were already familiar with their interviewer, so there was already an existing bond of trust between the interviewer and the interviewees, which could have led to more honest, but also to more socially desirable answers. Fourthly, interviews were conducted in six different languages, and ten interviews were conducted with the assistance of a translator by telephone. All interviews were transcribed and translated to English. In this process, words and intents may have changed in meaning and the data processing may have been influenced by divergent conceptualizations (Squires, 2009).

### Recommendation for Future Research and Practical Implications

We have discerned a total of 28 distinct strengths employed by refugees in their efforts to navigate stress, with a subset of seven strengths emerging as particularly salient in this context. This demonstrates that the VIA classification is a valuable instrument to identify personal strengths in a systematic way. However, it also shows a shortcoming of the instrument. Namely that solely using the VIA classification, strengths that may be more culturally bound or more relevant within the context of refugee wellbeing (e.g., patience, acceptance) could be overlooked. Therefore, we recommend that researchers investigating protective and resiliency factors among refugees utilize the VIA taxonomy and explore additional strengths using qualitative methods.

Results from this study may have implications for mental health care professionals working with refugees, and the specific focus of personal counselling and intervention programs. Firstly, most available intervention programs for refugees in the Netherlands aim to increase resilience, to reduce stress, or to develop psychosocial skills (Bloemen et al., 2021). Such programs often acknowledge the importance of developing personal strengths. Our study shows there is variety of specific strengths that may contribute to ability of refugees to cope with stress and help them to build up the skills they need to deal with the daily challenges in their lives. Future intervention or counselling programs could explicitly target the character strengths that are crucial in coping strategies of refugees that were identified in this study, or further identify essential character strengths for particular ethnic or cultural groups, as a part of the process of cultural adaptation. Secondly, we recommend that future programs shift from a problem-focused to a more strengths-based approach. This can be done, for example, by implementing the principles of the Aware-Explore–Apply strengths model (Niemiec, 2019). This is a three-step model, starting with making refugees aware of their own character strengths, followed by further exploration through reflection on past application and observing current use of strengths, and finally by discussing and implementing new ways for applying one’s strengths in practise. Thirdly, the shift from emotion-focused during the stay at an AZC to problem-focused coping when living in a Dutch municipality may have implications for programs that aim to increase mental well-being among refugees. Generally, mental health programs do not take refugees’ residential status into consideration and do not differentiate between the needs of refugees who live in a transition camp, an asylum center, or in the society of a host country. However, the motivational level and needs of refugees may vary according to the phase they are in, under what circumstances they are living, and the duration of their stay. Our study suggests that refugees who live at an AZC may gain more from intervention activities that help them regulate their emotions. Once they are living outside an AZC, refugees could benefit more from learning (psychological) skills that are required to attain set goals and solve practical problems. Therefore, the efficacy and acceptability of prevention and intervention programs could possibly be increased if the activities of such programs are framed within the context in which the programs are given.

## Conclusion

Previous research has identified religiousness and hope as individual characteristics that refugees use to cope with challenges in their daily life. The current study brings to light a wider range of character strengths that refugees apply, including self-regulation, perseverance, gratitude, and love of learning. Our study underlines the notion that there is a much broader spectrum of personal strengths that can function as protective factors and/or coping strategies. This offers starting points for enhancing well-being, instead of just alleviating suffering. By taking a positive psychological approach, we can learn more about the positive individual characteristics that are related to well-being or that protect people against psychological distress. Future mental health and social support programs could include more specific activities aimed at enhancing such individual strengths.

Furthermore, our study suggests that depending on the migration phase in which refugees find themselves, they use their character strengths for either emotion-focused or problem-focused coping. During their stay at an asylum center, strengths application is more related to emotion-focused coping, while once living in a municipality strengths use is more problem-focused. In addition, the migration phase may also influence the use of specific strengths. For example, whereas patience is particularly relevant in the context of coping with daily stress at an asylum center, perseverance is more relevant when coping with daily stressors once living in a municipality. Therefore, interventions that aim to increase mental well-being among refugees should also consider contextual factors such as the migration phase in which a refugee is situated, in addition to cultural factors. This may increase participant’s engagement in prevention and intervention programs, which could increase their acceptability and efficacy.

## Electronic Supplementary Material

Below is the link to the electronic supplementary material.

Supplementary Material 1

Supplementary Material 2

## Data Availability

The participants of this study did not provide consent for their data to be shared publicly. Due to the sensitive nature of the research, supporting data is not available.
