# Supplementary material for: Character Strengths as Coping Strategies for Daily Challenges: A Qualitative Study Among Adult Refugees
Source: Int J Appl Posit Psychol. 2025 Feb 8;10(1):24. doi: 10.1007/s41042-024-00211-z (PMC11954695; doi:10.1007/s41042-024-00211-z)
Supplement: Supplementary file 1 — Supplementary Material 1 [file 41042_2024_211_MOESM1_ESM.doc]

**Supplemental material 1. Overview of frequency of mentioned character strengths**

| **Character strength (VIA)** | **Inside AZC** | **In a municipality** | **Total** |
| --- | --- | --- | --- |
| Self-regulation | 16 | 9 | 25 |
| Perseverance | 6 | 13 | 19 |
| Gratitude | 8 | 10 | 18 |
| Love of Learning | 9 | 9 | 18 |
| Hope | 9 | 8 | 17 |
| Bravery | 3 | 7 | 10 |
| Honesty | 4 | 6 | 10 |
| Love | 4 | 6 | 10 |
| Prudence | 6 | 4 | 10 |
| Teamwork | 4 | 6 | 10 |
| Zest | 4 | 6 | 10 |
| Curiosity | 4 | 4 | 8 |
| Kindness | 5 | 3 | 8 |
| Creativity | 2 | 5 | 7 |
| Perspective | 4 | 3 | 7 |
| Humor | 3 | 2 | 5 |
| Fairness | 3 | 1 | 4 |
| Forgiveness | 1 | 3 | 4 |
| Modesty | 4 | 0 | 4 |
| Leadership | 0 | 4 | 4 |
| Social Intelligence | 3 | 1 | 4 |
| Spirituality | 0 | 2 | 2 |
| Appreciation of Beauty and Excellence | 1 | 0 | 1 |
| Judgment | 0 | 1 | 1 |
|  |  |  |  |
| **Character strengths (non-VIA)** | **Inside AZC** | **In a municipality** | **Total** |
| Patience | 16 | 5 | 21 |
| Adaptability | 13 | 6 | 19 |
| Acceptance | 3 | 7 | 10 |
| Harmony | 4 | 2 | 6 |
