# Supplementary material for: Character Strengths as Coping Strategies for Daily Challenges: A Qualitative Study Among Adult Refugees
Source: Int J Appl Posit Psychol. 2025 Feb 8;10(1):24. doi: 10.1007/s41042-024-00211-z (PMC11954695; doi:10.1007/s41042-024-00211-z)
Supplement: Supplementary file 2 — Supplementary Material 2 [file 41042_2024_211_MOESM2_ESM.doc]

**Supplemental material 2. Interview protocol**

**Preparation**

- Make sure you are in a room where you are not bothered by outside noise.
- Ensure the presence of tea/coffee/water and possibly biscuits.
- Test your audio recording function on the phone beforehand.

1. **Introduction**

- Welcome the participant and explain the purpose of the interview.

  *The purpose of this interview is to find out what well-being means for asylum seekers and what character strengths they use* *to cope with stress and challenges in their daily lives.

  Information from this interview is used to improve the BAMBOO program that strengthens the well-being of asylum seekers. With your answers you help to help other asylum seekers.*
- Go through the consent form with the participant and ensure that the form is signed twice. One copy is for the participant.
- If applicable: also explain to the interpreter, the purpose of the session and ask the interpreter to translate as much as possible simultaneously.
- Start the audio recording. Check whether the recording has started.
- Play the **Name and Fame game:**

  **Tell your name, what your name means, where the name comes from. Briefly include information about your family background and place of birth in your story. Then ask the participant to tell his/her story.**

1. **Well-being**

- Ask the following questions:

***What makes you happy?

What do you do every day to feel good?***

1. **Character strengths at the AZC**

- Explain what character strengths are:

  ***Character strengths are personal qualities that you have. They are properties that really belong to you and if you use, you feel you are.***
  Then give an example of one of your own character strengths and that of a family member to indicate the difference. Choose a quality that is NOT on one of the cards, so that you influence the participants as little as possible.
- Hand the deck of cards to the participant and indicate the following:
   ***I have 28 different cards here with different character strengths. Look at these cards. If you do not know exactly what a card means, you can look up what the card means in this list in front of you, or you can ask me.***After the participant has read the cards, indicate the following:
   ***I would like to ask you to choose 5 to 7 cards with character strengths that you think that AZC residents generally need to deal with the challenges in their life at the AZC.***
- After the participant has made the selection, discuss each selected card with him/her. Ask questions like:  ***Why did you choose this card?

  How does [selected quality] help an AZC resident to deal with the challenges of daily life at the AZC?***

1. **Character strengths outside the AZC**

- Collect the cards, shuffle them, and give the participant the following instruction.

***Suppose you will be living in your own house in a Dutch municipality in a few months, together with your family (if applicable). A new beginning with new challenges. Which character strengths will help you to deal with life outside the AZC. Pick 5 to 7 cards again.***

- After the participant has made the selection, discuss each selected card with him/her. Ask questions like:  ***Why did you choose this card?

  How does [selected quality] help to deal with the challenges in everyday life outside the AZC?***

1. **Closing**

- Thank the participant and give him/her the card set as a souvenir.
  Ask if he/she has any questions.
- Afterwards: scan the participation form and immediately make a copy of the audio recording.
